# Proteomic Profiling Change and Its Implies in the Early Mycosis Fungoides (MF) Using Isobaric Tags for Relative and Absolute Quantification (iTRAQ)

**DOI:** 10.1155/2020/9237381

**Published:** 2020-11-23

**Authors:** Mengyan Zhu, Yong Li, Cheng Ding, Jiaqi Wang, Yangyang Ma, Zhao Li, Xiaoyan Zhang, Ping Wang

**Affiliations:** ^1^The Third People's Hospital of Hangzhou Affiliated to Zhejiang Chinese Medical University, Hangzhou 310009, China; ^2^The Third People's Hospital of Hangzhou, Hangzhou 310009, China; ^3^Institute of Plant Physiology and Ecology, SIBS, CAS, Shanghai, China; ^4^Research Center, Shanghai Yeslab Biotechnology, Shanghai, China; ^5^People's Hospital of Cangzhou, Hebei, China

## Abstract

**Purpose:**

Mycosis fungoides (MF) is the most common T-cell lymphoma, with indolent biologic behavior in the early stage and features of invasive in the tumor stage. The diagnosis of MF is still ambiguous and difficult. We focused on the proteomic profiling change in the pathogenesis of early MF and identified candidate biomarkers for early diagnosis.

**Methods:**

We collected peripheral blood samples of MF patients and healthy individuals (HI) performed proteomic profiling analysis using isobaric tags for relative and absolute quantification (iTRAQ) platform. Differently expressed proteins (DEPs) were filtered, and involved biological functions were analyzed through Gene Ontology (GO) and Ingenuity Pathway Analysis (IPA) software.

**Results:**

We identified 78 DEPs including fifty proteins were upregulated and 28 proteins were downregulated in the MF group with HI as a control. Total DEPs were analyzed according to the biological regulation and metabolic process through GO analysis. The pathways of LXR/RXR activation and FXR/RXR activation were significantly activated, in which APOH, CLU, and ITIH4 were involved. The top annotated disease and function network was (Cancer, Organismal Injury and Abnormalities, Reproductive System Disease), with a key node CLU. These DEPs were involved in cancer, including thyroid carcinoma, head and neck carcinoma, and cancer of secretory structure, in which CLU, GNAS, and PKM played an indirect role in the occurrence and development of cancer. Relevant causal network was IL12 (family), which is related to GNAS, PKM, and other DEPs.

**Conclusion:**

Proteomic profiling of early-stage MF provided candidate protein biomarkers such as CLU, GNAS, and PKM, which benefit the early diagnosis and understanding of the mechanism of MF development. Besides, lipid metabolism may be one of the pathogenesis of MF, and IL12 was a potential marker for the diagnosis and treatment of early MF.

## 1. Introduction

Cutaneous T-cell lymphomas (CTCLs) originate from malignant T-lymphocytes, and their most common form is mycosis fungoides (MF), accounting for about 55% of cases [1]. Clinically, MF can be classified into three stages: patch, plaque, and tumor. Most of the cases show indolent biologic behavior. However, once there is a progressive skin lesion such as tumor, this disease will show significantly invasive biological behavior, including metastasis through peripheral blood, lymph nodes, or internal organs [2]. The early diagnosis and targeted therapy of MF are of real necessity. However, it is a great challenge to distinguish MF in the early stage from benign inflammatory skin diseases. Multifactors as heredity and environment play an essential role in the occurrence and development of MF, but the etiology and pathogenesis of MF have not been elucidated [3].

It is crucial to identify unique biomarkers of MF to assist the early diagnosis and monitor the response of therapy and prognosis. Different types of growth factors, cytokines, and chemokines are released in the bloodstream when skin changes or inflammation occurs. Evidence indicates that the tumor microenvironment plays an essential role in tumor development, not only in solid tumors but also in hematopoietic malignancies including MF [4, 5]. Chemokines, cytokines, adhesion molecules, and defective apoptosis that lead to skin homing of malignant T cells also promote the onset and progression of MF [4]. Analysis of the composition and dynamic changes of proteins in serum is an important method to directly evaluate the microenvironment of MF patients' cutaneous tissue and determine candidate biomarkers for diagnosis.

It is urgent to investigate protein-based biomarkers which can be widely used to improve the diagnosis and treatment of MF. Cowen et al. [6] successfully distinguished tumor stage (T3) MF, psoriasis, and healthy with acceptable accuracy by using serum proteomics. Multiple protein analysis tools have been used in protein identification and analysis in MF, such as surface-enhanced laser desorption/ionization-time-of-flight mass spectrometry (SELDI-TOF-MS) [6, 7] and two-dimensional gel electrophoresis (2-DE) [8]. SOD2, S100A8, FABP5, PARP-1, and IP-10 had been discovered and may be considered as a promising biomarker for the differentiation between MF and other dermatoses [7–9]. The iTRAQ technology has been used to analyze proteomics of tissues, fluids, and bacteria of animals and plants and made achievements in human diseases such as pancreatic cancer and glioma [10–13]. For the first time, we used iTRAQ technology to analyze the relationship of abundant serum proteins of early-stage MF patients, thus setting our sights on providing new biomarkers for diagnosis and pathogenesis of MF.

## 2. Materials and Methods

### 2.1. Patient and Sample Collection

Peripheral blood samples were collected from 9 mycosis fungoides (MF) and 9 healthy individuals (HI) in the Third People's Hospital of Hangzhou (Hangzhou, China). As controls, the age and sex of 9 HI were matched with the MF group. To improve results reliability, 9 samples of MF patients were randomly divided into three groups, and 3 samples were mixed within each group to obtain new three samples of T1, T2, and T3. By the same method, 9 control serum samples were randomly divided into three groups to obtain C1, C2, and C3 samples. All samples were collected with written informed consent using protocols that comply with the Declaration of Helsinki Principles. Permission for this study was obtained from the medical ethics committee (The Third People's Hospital of Hangzhou). The average age of 9 early-stage MF (5IA, 4IB) in this study, including 5 male cases and 4 female cases, is 43.77 years, and the course of disease ranged from 0.75 to 20 years with an average of 7.64 years, which was described in Supplementary Table S1. All patients were confirmed by clinical judgment, histopathology, immunophenotype detection, and/or T-cell receptor gene rearrangement analysis based on ISCL diagnostic algorithm for early MF published in 2005 [14]. None of the patients included in this research had other malignant tumors, and they were all adults without pregnancy.

### 2.2. Total Protein Extraction

The serum was separated from the blood after centrifugation. The frozen samples were treated with liquid nitrogen and fully ground in the liquid nitrogen environment. Then, 10% trichloroacetic acid was added at a ratio of 1 : 10 and placed it at -20°C for 1 hour. After centrifugation at 12000 rpm at 4°C for 15 minutes, the precipitate was treated with precooled acetone at -20°C for 1 hour and then repeated once. The sample was dried in vacuum for 10 minutes after centrifuge. After being dissolved in 1 ml protein extracted with protease inhibitor cocktail, the precipitate was intermittent sonication for 5 s, 10s off, a total of 80 cycles, followed by centrifuged to collect the supernatant. The protein contents were assayed using the Bradford assay; the remaining samples were stored at -80°C.

### 2.3. Protein Preparation for iTRAQ

Proteins of 100 *μ*g for each sample were washed with 1 : 5 water : acetone to fully precipitate protein at -20°C for 1 hour. Centrifugated and vacuum-dried protein precipitates were fully dissolved in dissolution buffer in the iTRAQ kit (AB SCIEX, Sigma), then reduced to alkylation at 60°C for 1 hour after 4 *μ*l reducing reagent was added. Ultrafiltration and centrifugation of the reduced alkylated protein solution were performed after reacting with 2 *μ*l cysteine-blocking reagent at room temperature for 10 minutes, and then the ITRAQ reagent was used to label proteins of MF samples and HIs samples which were digested with trypsin for 12 hours at 37°C. All labeled samples were mixed and centrifuged in a tube, then vacuum freeze-dried for iTRAQ separation and identification.

### 2.4. Mass Spectrometry Assay

The freeze-dried samples were dissolved in 100 *μ*l buffer A solution and proceeded SCX separation using nanoflow CHIP-LC (CHIP-nLC, AB Sciex). The samples were first transported to Dionex Acclaim PepMap100 C18 nanoliter reverse column (5 *μ*m, 100 A, 100 *μ*m I.D.X 2 cm, Thermo Fisher Scientific, Sunnyvale, CA, USA) for analysis, whose loading volume was 10 *μ*l and the flow rate was set to 350 nl/min. Then, the peptides were separated on RSLC C18 chromatographic column (2 *μ*m, 100A, 75 m microns I.D.X 25 cm, Thermo Fisher Scientific, Sunnyvale, CA, USA) whose mobile phase A was 2%ACN+0.5% acetic acid, and mobile phase B was 98% ACN+0.5% acetic acid according to the gradient. The elution program was as follows: 10 min, 4-9% A; 100 min, 9-33% A; 30 min, 33-50% A; 10 min, 50-100% A; 10 min, 100% B; and 10 min, 95% A. The eluted peptide was sprayed into the mass spectrometer (TripleTOF mass spectrometer, AB Sciex) at a voltage of 1.2 kV. The most abundant 30 polypeptides dynamically determined from the first column mass spectrum entered the second column implement relative collision-induced dissociation (CID).

### 2.5. Identification and Analysis of Proteins

After the samples were analyzed by mass spectrometry, we applied the search engine, Mascot server (version 2.3, Matrix Science, London, UK) to retrieve the data in the protein database. All results were filtered to summarize unique peptides according to FDR (<0.01). Meanwhile, Target Decoy PSM Validator and the expected value of Mascot in Proteome Discover were used to verify the search results. Only the peptide identification results meet both FDR ≤ 0.01 and *P* ≤ 0.01 were considered feasible. The following options were used to identify the proteins: fixed modifications: acrylamide modification on cysteine, acetylation modification on lysine and peptide N-terminal, acetamide modification on aspartate and glutamate, and dimethyl modification on peptide N-terminal; cutting mode: enzyme is trypsin and the maximum missed cut point is 2; and variable modification: acetamide modification at the C terminal of the protein and the oxidation of methane glycine. Differentially expressed proteins (DEPs) were filtered according to the criteria: *P* value is less than 0.05 and fold change > 1.3.

### 2.6. Protein Function Analysis

Gene Ontology (GO) is an important method and tool to represent biological knowledge in the field of biological information [15]. The bioinformatics analysis tool WebGestalt (http://www.webgestalt.org) was used to perform GO analysis [16]. Networks and functional analysis were generated using Ingenuity Pathway Analysis (IPA) (http://www.ingenuity.com), which is an up-to-date, web-based biological causal analysis approaches, can be used to get the functions of genes, proteins, and chemicals and their relevant biological pathways, regulators, and networks [17]. All DEPs were analyzed using the IPA software.

## 3. Results

### 3.1. Analysis of Proteins and Peptides

**Figure 1:**
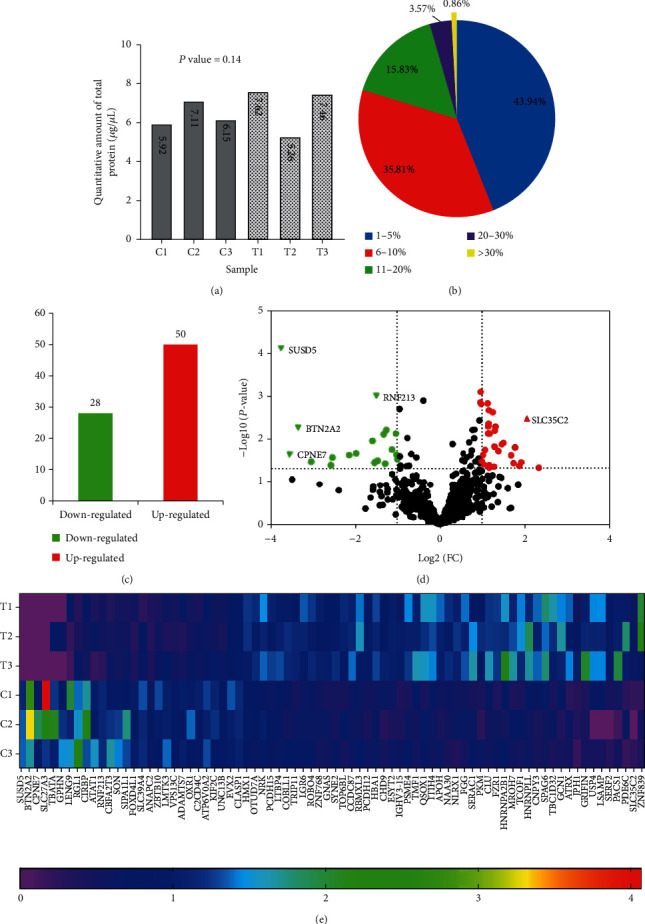
Serum proteome profiling analysis using iTRAQ technology. (a) Quantitative of total protein in each sample; (b) Distribution of peptide sequence coverage; (c) Differentially expressed proteins (DEPs) in each group; (d) Volcanic plot displayed DEPs, upper left represented downregulated proteins and upper right represented upregulated proteins; (e) Heatmap of DEPs between MF and HIs.

The total protein of each sample ranged from 5.28 to 7.46 *μ*g/*μ*l by Bradford assay was used to assay (Figure 1(a)), and there was no significant difference between the control group and the patient group (*P* = 0.140). After the protease cleavage products labeled with iTRAQ, proteins were analyzed by mass spectrometry and Mascot server, and the results were merged and filtered by peptide FDR ≤ 0.01. We found that 16,039 unique peptides were selected from 26,938 peptides split from 701 proteins. Nearly 80% of the protein's sequence coverage is below 10%, close to 50% is below 5% (Figure 1(b)) in the identified proteins.

We filtered and found 78 DEPs according to the cutoff value of 1.3 fold change and *P* value < 0.05. Fifty proteins were upregulated, and 28 proteins were downregulated (Figure 1(c)). The volcanic plot graph showed DEPs located in the upper left and upper right (Figure 1(d)), and Figure 1(e) is the heat map of DEPs between MF and HIs. Detailed information of DEPs was provided in Supplementary Table S2, and Table 1 is the top 20 upregulated and downregulated proteins.

### 3.2. Gene Ontology (GO) Analysis of Differentially Expressed Proteins

**Figure 2:**
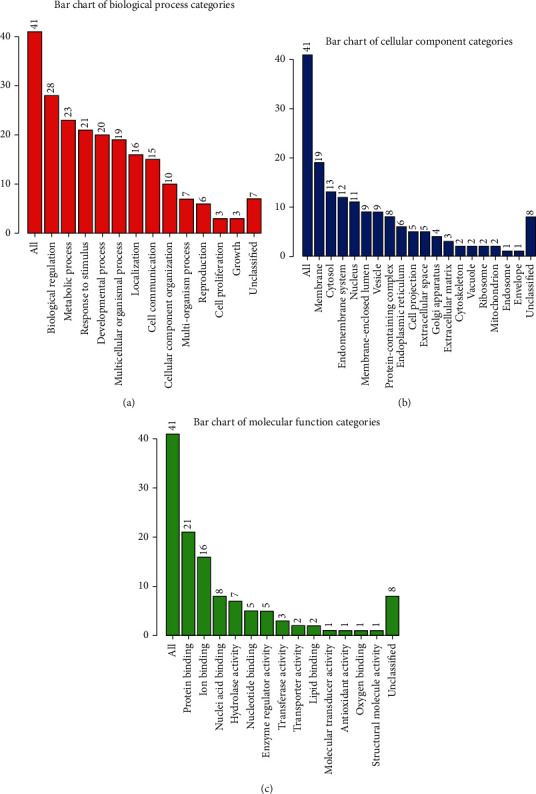
GO enrichment of differently expressed proteins (DEPs) between MF and HI samples. (a) Biological process enrichment of DEPs. (b) The enrichment of cellular component in DEPs. (c) The molecular function analysis of DEPs.

We performed the GO analysis on 78 proteins with 3 major clusters of biological process, cellular components, and molecular function to predict their possible roles in MF development. The Uniport ID of DEPs inputted into WebGestalt for annotation analysis. The GO slim summaries were based upon the 41 unique IDs that unambiguously mapped from 78 DEPs. DEPs including upregulated and downregulated proteins are classified according to their molecular function, cellular component, and biological function using WebGestalt (Figure 2).

Viewed from the biological process, the top 3 enrichment items were biological regulation, metabolic process, and response to stimulus, and each of them was accounted for over 50% (Figure 2(a)). In the level of cellular component, membrane, cytosol, and endomembrane system and nucleus occupied a large proportion (Figure 2(b)). For molecular function classification, the differently expressed proteins were mainly involved in protein binding, ion binding, and nucleic acid binding in this part (Figure 2(c)).

### 3.3. Analysis of Canonical Pathways and Upstream Regulators Involved in DEPs

**Figure 3:**
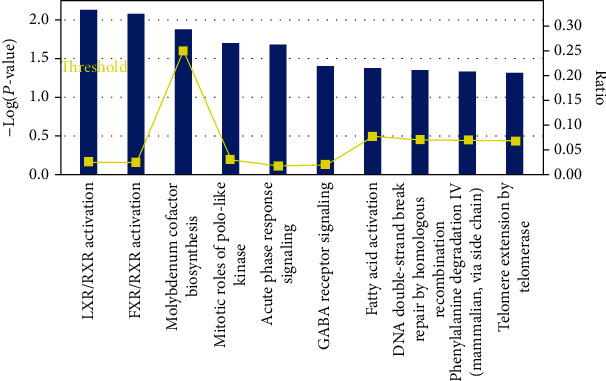
Top canonical pathways based on 78 differentially expressed proteins (DEPs).

The canonical pathways involved in 78 DEPs were analyzed by IPA, and the illustration of top canonical pathways was displayed in Figure 3. The pathways of LXR/RXR activation, FXR/RXR activation, molybdenum cofactor biosynthesis, mitotic roles of Polo-like kinase, and acute phase response signaling were descripted, which were described in Supplementary Table S3. LXR/RXR activation and FXR/RXR activation were activated, and APOH, CLU, and ITIH4 were involved in these two pathways. GPHN protein downregulation in the MF group which is involved in the pathway of activated molybdenum cofactor biosynthesis.

The upstream regulators of differently expressed proteins were analyzed by IPA, which included HNRNPA1, recombinant interferon alpha, PDE6H, cevimeline, AIPL1, MDC1, and clusterin antisense oligonucleotide (Supplementary Table S4). Both of MDC1 and clusterin antisense oligonucleotide upstream factors are related to the target protein CLU. The interactive relationships between upstream regulators and their target proteins in the dataset were displayed in the network (Supplementary Figure S1).

### 3.4. Related Diseases and Function Networks

**Figure 4:**
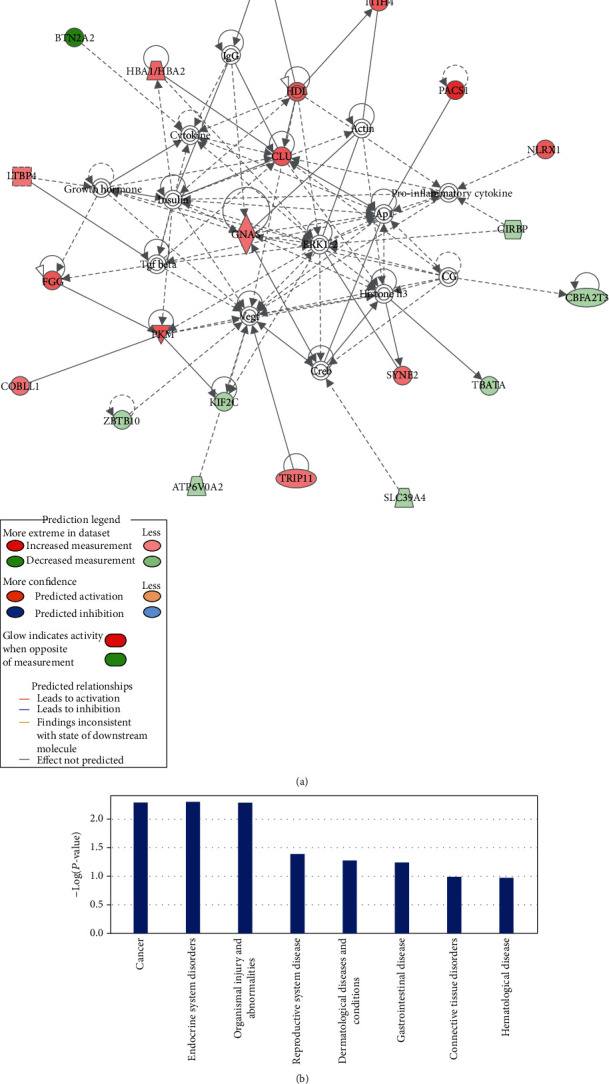
Graph of regulation network and related diseases involved in DEPs: (a) Network of (Cancer, Organismal Injury and Abnormalities, Reproductive System Disease); (b) The categories of the network of cancer.

The disease and function network obtained by IPA analysis have (Cancer, Organismal Injury and Abnormalities, Reproductive System Disease), (Hematological System Development and Function, Hematopoiesis, Tissue Morphology), (Cancer, Endocrine System Disorders, Neurological Disease), (Cell Morphology, Nucleic Acid Metabolism, Small Molecule Biochemistry), (Cancer, Dermatological Diseases and Conditions, Organismal Injury and Abnormalities), and (Cancer, Endocrine System Disorders, Gastrointestinal Disease), most of which were related to tumors (Supplementary Table S5). The top-ranked network is (Cancer, Organismal Injury and Abnormalities, Reproductive System Disease) (Figure 4(a)). APOH, ITIH4, PACS1, NLRX1, HDL, HBA1/HBA2, CLU, GNAS, LTBP4, FGG, PKM, COBLL1, SYNE2, and TRIP11 were upregulated in the MF group, while BTN2A2, CIRBP, and CBFA2T3 were downregulated in MF. We can find CLU is a key node in this network. As can be seen from the figure, a variety of proteins can directly or indirectly activate the expression of CLU, such as CLU self-activated and activating CLU through increased expression of HDL.

IPA analysis found that the disease or disorder most associated with 78 DEPs involved in cancer, including thyroid carcinoma, head and neck carcinoma, and cancer of secretory structure (Supplementary Table S6), and it can be directly seen that they are related to dermatological diseases and conditions (Figure 4(b)). The study on incidence of tumor and development of carcinoma found that some DEPs indirectly inhibited the occurrence and development of MF, including CLU, GNAS, FZR1, and PKM (Supplementary Figure S2, Table 2).

**Figure 5:**
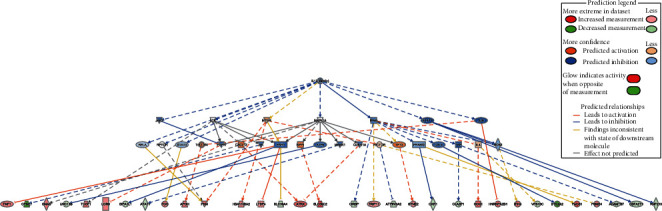
IL12 (family) causal network.

The top causal network mainly covered IL12 (family), genistein, HIP1, MCF2, and SRC (Supplementary Table S7). The most relevant causal network was IL12 (family), whose network of DEPs is shown below (Figure 5). TMF1, GNAS, TCOF1, LGR6, FGG, APOH, PKM, HBA1/HBA2, LTBP4, ATRX, SLC35C2, TRIP11, SYNE2, GCN1, HNRNPA2B1, ITIH4, PACS1, and PSME4 were upregulated, while CPNE7, UNC13B, SIPA1L1, ATAT1, SLC39A4, CIRBP, ATP6V0A2, CXR1, CLASP1, VPS13C, BTN2A2, ADAMTS7, CBFA2T3, and RNF213 were downregulated. IL12 regulates downstream proteins through directly inhibiting IFNG and indirectly inhibiting JAK2, LCK, MAPK14, STAT1, and JUN, but MTOR is found inconsistent with the stage of downstream molecule. JAK2, MAPK14, STAT1, and JUN were predicted inhibition.

Protein CLU's canonical pathways include LXR/RXR activation and FXR/RXR activation, GNAS is related to GABA receptor signaling pathway, and PKM is closely connected with glycolysis I pathway. Whereas three upexpression proteins are all most likely associated with (Cancer, Organismal Injury and Abnormalities, Reproductive System Disease) network. Moreover, there is a close connection between them and cancer (thyroid carcinoma, head and neck carcinoma, cancer of secretory structure, incidence of tumor, and development of carcinoma). GNAS and PKM are both regulated by IL12 (family), while CLU and PKM are related to the genistein network.

## 4. Discussion

The proteins IP-10, SOD2, S100A8, FABP5, and PARP-1 have been identified as potential biomarkers for MF in previous studies [7–9, 13]. In the other hand, CD26 soluble serum levels and the expression of TOX, Tplastin, TWIST, CD158, and nkP46 may contribute to the differential diagnosis of MF [18–22]. In our study, we identified 50 upregulated and 28 downregulated proteins in MF. IPA analysis indicated that pathways of LXR/RXR activation and FXR/RXR activation were most significantly activated, in which APOH, CLU, and ITIH4 were involved in cancer, organismal injury and abnormalities, and reproductive system disease. CLU, GNAS, and PKM played key nodes in the occurrence and development of cancer. And they are also involved in relevant causal network IL12 (family). Based on the above proteomic profiling results, it is speculated that CLU, GNAS, and PKM are more likely to play a crucial role in the direction of MF and maybe are new biomarkers for diagnosis and pathogenesis of MF.

Liver X receptors/Retinoid X receptor (LXR/RXR) and Farnesoid X receptor/Retinoid X receptor (FXR/RXR) activation pathways are closely related to lipid and glucose metabolism, cholesterol transport, bile acid homeostasis, and the modulation of inflammatory responses [23–25]. Inhibition of the LXR/RXR and FXR/RXR pathway will lead to intracellular lipid accumulation and ultimately to cell apoptosis and inflammation, called lipotoxicity [26–28]. Though it still needs more extensive population-based studies, researchers have referred that patients with MF had significantly higher levels of total cholesterol compared with age and sex-matched control group, and cardiovascular disease may be one of the risk factors in MF [29, 30]. Lipid antigens stimulate T cells through cluster differentiation 1 molecules (CD1) predominantly by dendritic cells and B cells [31]. Increased numbers of CD1a- and CD1c-expressing dendritic cells have been reported previously in MF and folliculotropic mycosis fungoides (FMF) [32–34], supporting a role that lipid may be an etiology of MF. Secretory clusterin (sCLU), one of Clusterin (CLU) categories, also known as apolipoprotein J (apoJ), participates in lipid transport [35] and is considered as a protective factor in vascular disease progression [29]. We found that the expression of CLU was upregulated in the early stage of MF which is involved in the LXR/RXR and FXR/RXR pathways (Supplementary Table S3). The high expression of CLU will inhibit two pathways, thus increasing the effects of endoplasmic reticulum stress, mitochondrial dysfunction, oxidative stress, and defective intracellular signaling caused by intracellular lipid accumulation, and thereby aggravating the apoptosis of cells [28]. In our study, these two pathways that serve as a pathway for the development of early MF were upregulated.

The role of CLU in tumor is still controversial. For example, the plasma CLU level of prostate cancer and prostatic hyperplasia is higher than that of normal prostate, which was positively correlated with pathological grade and stage [36]. Rizzi et al. [37] found that CLU mRNA is significantly downregulated in prostate cancer tissue at early stages compared to normal prostate. The studies confirmed the hypothesis that CLU could indeed act as a tumor suppressor gene by knockout of the CLU gene [38, 39]. As we can see in Table 2, CLU indirectly inhibited the incidence and development of tumor. Therefore, we believe that the role of CLU should be divided into stages which play a role in tumor suppressor genes in the early stage but promotes the development of cancer in the later stage when the tumor suppressor factor is inactivated or acquiring improper activity like pRb [40]. Olsen et al. [41] disclosed clusterin expression in 75% of MF cases with large cell transformation (MF-LCT). Another study followed that 105 cases of MF and Sézary syndrome's tissue sections were immunostained for clusterin [42]. Clusterin was positive in 13 (12.4%) cases, of which 6 were early T-stage, 3 were MF-LCT positive in the initial diagnostic biopsy, and interestingly, 5 positive cases subsequently developed LCT suggesting that CLU may be a novel unfavorable prognostic marker for MF. In our study, the expression of CLU increased, which may be due to MF as an inert tumor, CLU plays an inhibitory role on MF in the early stage, and on the other hand, it indicates the prognosis of the disease. However, whether CLU expression increases in early MF patients still requires a larger number of samples with a wider age range to prove.

The *gnas* gene encodes multiple gene products, of which the most abundant and best-characterized one is the alpha subunit of the stimulatory guanine nucleotide-binding protein (Gsɑ). Gsɑ is a signaling protein needed for the actions of numerous hormones, neurotransmitters, and autocrine/paracrine factors [43]. The second messenger generated by Gsɑ, cAMP, effectively inhibits the proliferation and progression of tumor cells. Based on this principle, Gsɑ has been found to be the tumor suppressor in Sonic Hedgehog-driven Medulloblastoma [44]. When parathyroid hormone (PTH) binds to its receptor, Gsa is dissociated through the Gs*α*/cAMP/PKA signaling pathway [45]. PTH refers to rare clinical and endocrine manifestations like ectopic ossification, TSH resistance, GH deficiency, and early-onset obesity. This is consistent with IPA analysis results that 78 DEPs including GNAS were closely related to thyroid carcinoma and cancer of secretory structure (Supplementary Table S6). And in previous studies, *gnas* mutation has been found in various tumors, including those of endocrine, gastrointestinal, lung, and acute leukemia, with ocular melanoma and appendiceal cancer having the strongest associations [46, 47]. The GNAS^R201C^ mutation may maintain lymphoid-biased hematopoietic stem cells at a developmental state favorable for transformation [48]. Besides, the T393C polymorphism of GNAS is associated with increased Gsɑ mRNA expression in solid tumors and in chronic lymphocytic leukemia (CLL) [49]. In conclusion, the increase of GNAS expression is related to MF disease to some extent. The specific mechanism is unknown and needs further study, which may be related to Gsɑ.

In addition, we found that the expression of Pyruvate kinase M (PKM) was upregulated in MF (Supplementary Table S2). PKM is a critical factor involved in glycolysis, mainly including these two isoforms PKM1 and PKM2, and especially PKM2 expression is highly elevated in human tumors [50]. PKM2 has a critical dual role in proliferating and quiescence endothelial cells [51], but PKM1 cannot promote tumor development; in consequence, replacing PKM2 with PKM1 has been shown to inhibit aerobic glycolysis and tumor growth [52]. Activating PKM2 to get PKM1-like activity may be a better way to treat cancer than inhibiting PKM2 [53, 54]. Meanwhile, studies have proved that TEPP-46, as an activator of PKM2 pyruvate activity, mimics the PKM1 properties in PKM2-expressing cells, therefore inhibits cancer cell proliferation in vitro and xenograft tumor growth in vivo [53, 55]. This suggests that the increase of PKM expression in this study was significantly correlated with the occurrence and development of MF.

The level of IL-12 is increased in the patch stage of MF, so some researchers believe that IL-12 immunohistochemistry can be a diagnostic tool [56]. Previous studies have investigated using recombinant interleukin-12 (rhIL-12) in the treatment of MF. The phase I and phase II trials using rhIL-12 for CTCLs on 10 and 32 evaluable patients, respectively, have demonstrated the activity of IL-12 with an overall response rate of about 50% [56, 57]. According to our IPA analysis (Supplementary Table S7 and Figure 5), the top causal network is also IL-12 (family), GNAS and PKM are significantly correlated with IL-12, which promotes the expression of the Th1 phenotype to improve the Th1/Th2 imbalance in MF [58, 59]. The above results indicate that IL-12 has more potential value in early MF diagnosis and treatment.

In summary, these results demonstrated that CLU, GNAS, and PKM are differentially expressed in the serum of MF patients and normal subjects, which may inhibit the occurrence and development of tumor directly/indirectly. It speculated that the upregulated expression of CLU, GNAS, and PKM may inhibit the occurrence of tumor in the early stage, while the expression decreased in the late-stage promotes the development of tumor. CLU is in a crucial part of the LXR/RXR and FXR/RXR activation pathways, so the increase of CLU content in serum suggests that the pathogenesis of MF may be related to lipid metabolism. However, this conclusion needs to be verified by more early and tumor stage clinical samples, and their function in MF also needs to be further studied. IL-12 as a corelated factor of the two proteins plays an important role in the pathogenesis of MF. IL12 can be used not only as a marker for diagnosing MF in early-stage which changes in the progression but also for disease treatment. On the basis of previous studies, this research adds an important basis for the role of IL-12 in the diagnosis and treatment of MF.

## Figures and Tables

**Table 1:** The top 20 upregulated and downregulated differentially expressed protein (DEPs).

Protein name	Accession	Description	MF group	HI group	Old change	Down/up
SUSD5	H7C2K7	Sushi domain-containing protein 5	0.000	1.311	0.000	Down
BTN2A2	Q8WVV5	Butyrophilin subfamily 2 member A2	0.000	2.667	0.000	Down
CPNE7	H0YEH8	Copine-7	0.000	1.242	0.000	Down
SLC27A3	X6R3N0	Long-chain fatty acid transport protein 3	0.000	2.667	0.000	Down
TBATA	Q96M53	Protein TBATA	0.259	1.504	0.172	Down
GPHN	Q9NQX3	Gephyrin	0.214	1.213	0.176	Down
LENG9	Q96B70	Leukocyte receptor cluster member 9	0.370	1.598	0.231	Down
RGL1	Q9NZL6	Ral guanine nucleotide dissociation stimulator-like 1	0.478	1.850	0.258	Down
CIRBP	Q14011-3	Isoform 3 of cold-inducible RNA-binding protein	0.598	1.778	0.336	Down
ATAT1	Q5SQI0-6	Isoform 6 of alpha-tubulin N-acetyltransferase 1	0.416	1.196	0.347	Down
ATRX	P46100-2	Isoform 1 of transcriptional regulator ATRX	1.281	0.453	2.829	Up
JPH1	Q9HDC5	Junctophilin-1	0.810	0.253	3.207	Up
GRIFIN	A4D1Z8	Grifin	1.456	0.437	3.333	Up
USP4	Q13107	Ubiquitin carboxyl-terminal hydrolase 4	1.372	0. 401	3.418	Up
LSAMP	F5H5G1	Limbic system-associated membrane protein	1.378	0.403	3.418	Up
SERF2	C9JQZ0	Small EDRK-rich factor 2 (fragment)	0.824	0.241	3.418	Up
PACS1	Q6VY07	Phosphofurin acidic cluster sorting protein 1	1.379	0.378	3.654	Up
PDE6C	P51160	Cone cGMP-specific 3′ 5′-cyclic phosphodiesterase subunit alpha′	1.303	0.345	3.773	Up
SLC35C2	Q5JW04	Solute carrier family 35 member C2	0.600	0.145	4.144	Up
ZNF839	A8K0R7	Zinc finger protein 839	1.814	0.362	5.005	Up

**Table 2:** Differentially Expressed Proteins associated with the incidence and development of cancer.

Differentially expressed proteins	Role in the incidence and development of cancer
CLU, GNAS, PKM, FZR1	Indirect inhibition of the incidence of tumor and development of carcinoma
LGR6, UNC138	Indirect inhibition of the incidence of tumor
ITIM4	Findings inconsistent with state of downstream
ZNF839, USP4, LSAMP, PACS1, SERF2, JPH1, ATRX, BTN2A2, CPNE7, SUSD5, SLC27A3, etc.	Effect not predicted

## Data Availability

All raw data in this article can be obtained by emailing the corresponding author.

## Supplementary Materials

Supplementary Materials Supplementary Table S1: clinical data of MF patients. Supplementary Table S2: the analysis of differentially expressed protein. Supplementary Table S3: detailed top 5 canonical pathways. Supplementary Table S4: top upstream regulators. Supplementary Table S5: top network. Supplementary Table S6: the categories of the network of cancer. Supplementary Table S7: top causal network. Supplementary Figure S1: the interactive relationships between upstream regulators and target proteins
