# Supplementary material for: Proteomic Profiling Change and Its Implies in the Early Mycosis Fungoides (MF) Using Isobaric Tags for Relative and Absolute Quantification (iTRAQ)
Source: Biomed Res Int. 2020 Nov 23;2020:9237381. doi: 10.1155/2020/9237381 (PMC7707953; doi:10.1155/2020/9237381)
Supplement: Supplementary Materials — Supplementary Table S1: clinical data of MF patients. Supplementary Table S2: the analysis of differentially expressed protein. Supplementary Table S3: detailed top 5 canonical pathways. Supplementary Table S4: top upstream regulators. Supplementary Table S5: top network. Supplementary Table S6: the categories of the network of cancer. Supplementary Table S7: top causal network. Supplementary Figure S1: the interactive relationships between upstream regulators and target proteins [file 9237381.f1.docx]

Supplementary Table S1. Information of MF patients.

| Patients | Gender | Age（years） | Sick time（years） | Clinically suspected | Clinical manifestation | Stage |
| --- | --- | --- | --- | --- | --- | --- |
| MF1 | Female | 55 | 20 | MF | The whole body erythema | ⅠB |
| MF2 | Female | 30 | 0.75 | MF | The whole body erythema | ⅠA |
| MF3 | Female | 52 | 10 | MF | Lower limbs erythema | ⅠA |
| MF4 | Female | 39 | 20 | MF | The whole body erythema | ⅠB |
| MF5 | Male | 52 | 5 | MF | The trunk and limbs erythema | ⅠA |
| MF6 | Male | 52 | 2 | MF | The whole body erythema | ⅠB |
| MF7 | Male | 40 | 1 | MF | The trunk erythema | ⅠA |
| MF8 | Male | 40 | 6 | MF | The trunk erythema | ⅠA |
| MF9 | Male | 34 | 4 | MF | The whole body erythema | ⅠB |

Supplementary Table S2 The analysis of differentially expressed protein

| Protein name | Accession | Description | Av.T1/2/3 | Av.C1/2/3 | P value | Fold change | Up/  down |
| --- | --- | --- | --- | --- | --- | --- | --- |
| **SUSD5** | **H7C2K7** | **Sushi domain-containing protein 5** | **0.000** | **1.311** | **0.000** | **0.000** | **Down** |
| **BTN2A2** | **Q8WVV5** | **Butyrophilin subfamily 2 member A2** | **0.000** | **2.667** | **0.006** | **0.000** | **Down** |
| **CPNE7** | **H0YEH8** | **Copine-7** | **0.000** | **1.242** | **0.024** | **0.000** | **Down** |
| **SLC27A3** | **X6R3N0** | **Long-chain fatty acid transport protein 3** | **0.000** | **2.667** | **0.035** | **0.000** | **Down** |
| **TBATA** | **Q96M53** | **Protein TBATA** | **0.259** | **1.504** | **0.042** | **0.172** | **Down** |
| **GPHN** | **Q9NQX3** | **Gephyrin** | **0.214** | **1.213** | **0.028** | **0.176** | **Down** |
| **LENG9** | **Q96B70** | **Leukocyte receptor cluster member 9** | **0.370** | **1.598** | **0.024** | **0.231** | **Down** |
| **RGL1** | **Q9NZL6** | **Ral guanine nucleotide dissociation stimulator-like 1** | **0.478** | **1.850** | **0.022** | **0.258** | **Down** |
| **CIRBP** | **Q14011-3** | **Isoform 3 of Cold-inducible RNA-binding protein** | **0.598** | **1.778** | **0.011** | **0.336** | **Down** |
| **ATAT1** | **Q5SQI0-6** | **Isoform 6 of Alpha-tubulin N-acetyltransferase 1** | **0.416** | **1.196** | **0.037** | **0.347** | **Down** |
| RNF213 | Q63HN8 | E3 ubiquitin-protein ligase RNF213 | 0.428 | 1.191 | 0.001 | 0.359 | Down |
| CBFA2T3 | O75081-4 | Isoform 3 of Protein CBFA2T3 | 0.474 | 1.295 | 0.033 | 0.366 | Down |
| SON | P18583-10 | Isoform J of Protein SON | 0.501 | 1.241 | 0.008 | 0.404 | Down |
| SIPA1L1 | O43166-3 | Isoform 3 of Signal-induced proliferation-associated 1-like protein 1 | 0.558 | 1.353 | 0.039 | 0.413 | Down |
| FOXD4L1 | Q9NU39 | Forkhead box protein D4-like 1 | 0.404 | 0.960 | 0.006 | 0.421 | Down |
| SLC39A4 | E9PQ16 | Zinc transporter ZIP4 | 0.576 | 1.249 | 0.018 | 0.461 | Down |
| ANAPC2 | Q9UJX6-2 | Isoform 2 of Anaphase-promoting complex subunit 2 | 0.334 | 0.681 | 0.008 | 0.491 | Down |
| ZBTB10 | Q96DT7-2 | Isoform 2 of Zinc finger and BTB domain-containing protein 10 | 0.539 | 1.084 | 0.024 | 0.497 | Down |
| LMTK3 | Q96Q04 | Serine/threonine-protein kinase LMTK3 | 0.599 | 1.190 | 0.031 | 0.504 | Down |
| VPS13C | Q709C8-4 | Isoform 4 of Vacuolar protein sorting-associated protein 13C | 0.464 | 0.890 | 0.002 | 0.521 | Down |
| ADAMTS7 | Q9UKP4 | A disintegrin and metalloproteinase with thrombospondin motifs 7 | 0.522 | 0.997 | 0.026 | 0.523 | Down |
| OXR1 | Q8N573-2 | Isoform 2 of Oxidation resistance protein 1 | 0.582 | 1.100 | 0.043 | 0.529 | Down |
| C2CD4C | Q8TF44 | C2 calcium-dependent domain-containing protein 4C | 0.540 | 0.926 | 0.044 | 0.583 | Down |
| ATP6V0A2 | Q9Y487 | V-type proton ATPase 116 kDa subunit a isoform 2 | 0.742 | 1.255 | 0.010 | 0.591 | Down |
| KIF2C | Q99661-2 | Isoform 2 of Kinesin-like protein KIF2C | 0.674 | 1.108 | 0.043 | 0.608 | Down |
| UNC13B | A0A1B0GUS7 | Protein unc-13 homolog B | 0.544 | 0.863 | 0.023 | 0.630 | Down |
| EVX2 | Q03828 | Homeobox even-skipped homolog protein 2 | 0.789 | 1.154 | 0.028 | 0.684 | Down |
| CLASP1 | Q7Z460-5 | Isoform 5 of CLIP-associating protein 1 | 0.899 | 1.175 | 0.765 | 0.001 | Down |
| HMX1 | Q9NP08 | Homeobox protein HMX1 | 1.174 | 0.818 | 0.032 | 1.435 | Up |
| OTUD7A | Q8TE49 | OTU domain-containing protein 7A | 1.146 | 0.783 | 0.022 | 1.463 | Up |
| NRK | Q7Z2Y5 | Nik-related protein kinase | 1.361 | 0.904 | 0.035 | 1.506 | Up |
| PCDH15 | Q96QU1 | Protocadherin-15 | 1.116 | 0.714 | 0.029 | 1.563 | Up |
| LTBP4 | Q8N2S1-3 | Isoform 3 of Latent-transforming growth factor beta-binding protein 4 | 1.150 | 0.707 | 0.013 | 1.627 | Up |
| COBLL1 | Q53SF7-2 | Isoform 2 of Cordon-bleu protein-like 1 | 1.146 | 0.686 | 0.025 | 1.670 | Up |
| TRIP11 | Q15643 | Thyroid receptor-interacting protein 11 | 1.021 | 0.611 | 0.045 | 1.671 | Up |
| LGR6 | Q9HBX8 | Leucine-rich repeat-containing G-protein coupled receptor 6 | 1.163 | 0.684 | 0.020 | 1.699 | Up |
| ROBO4 | Q8WZ75-2 | Isoform 2 of Roundabout homolog 4 | 1.150 | 0.668 | 0.007 | 1.721 | Up |
| ZNF768 | H3BS42 | Zinc finger protein 768 | 0.988 | 0.559 | 0.010 | 1.767 | Up |
| GNAS | P84996 | Protein ALEX | 0.944 | 0.532 | 0.006 | 1.773 | Up |
| SYNE2 | A0A0A0MRE3 | Nesprin-2 | 0.997 | 0.553 | 0.035 | 1.802 | Up |
| TOP6BL | Q8N6T0-4 | Isoform 1 of Type 2 DNA topoisomerase 6 subunit B-like | 1.059 | 0.558 | 0.030 | 1.898 | Up |
| CCDC87 | Q9NVE4 | Coiled-coil domain-containing protein 87 | 1.236 | 0.644 | 0.004 | 1.921 | Up |
| RBMXL3 | Q8N7X1 | RNA-binding motif protein X-linked-like-3 | 1.349 | 0.698 | 0.038 | 1.933 | Up |
| PCDH12 | Q9NPG4 | Protocadherin-12 | 0.982 | 0.503 | 0.002 | 1.952 | Up |
| HBA1 | P69905 | Hemoglobin subunit alpha | 1.270 | 0.648 | 0.001 | 1.959 | Up |
| CHD9 | Q3L8U1-2 | Isoform 2 of Chromodomain-helicase-DNA-binding protein 9 | 0.890 | 0.454 | 0.035 | 1.961 | Up |
| ESYT2 | A0FGR8 | Extended synaptotagmin-2 | 1.062 | 0.537 | 0.002 | 1.978 | Up |
| IGHV3-15 | A0A0B4J1V0 | Immunoglobulin heavy variable 3-15 | 0.905 | 0.455 | 0.034 | 1.992 | Up |
| PSME4 | Q14997 | Proteasome activator complex subunit 4 | 1.277 | 0.627 | 0.025 | 2.036 | Up |
| TMF1 | P82094 | TATA element modulatory factor | 1.288 | 0.626 | 0.041 | 2.058 | Up |
| QSOX1 | O00391-2 | Isoform 2 of Sulfhydryl oxidase 1 | 1.485 | 0.705 | 0.019 | 2.105 | Up |
| ITIH4 | Q14624 | Inter-alpha-trypsin inhibitor heavy chain H4 | 1.471 | 0.670 | 0.002 | 2.196 | Up |
| APOH | P02749 | Beta-2-glycoprotein 1 | 1.187 | 0.537 | 0.008 | 2.212 | Up |
| NAA30 | Q147X3-2 | Isoform 2 of N-alpha-acetyltransferase 30 | 1.135 | 0.513 | 0.005 | 2.213 | Up |
| NLRX1 | Q86UT6 | NLR family member X1 | 1.159 | 0.519 | 0.005 | 2.230 | Up |
| FGG | P02679-2 | Isoform Gamma-A of Fibrinogen gamma chain | 1.310 | 0.585 | 0.002 | 2.240 | Up |
| SERAC1 | Q96JX3 | Protein SERAC1 | 1.357 | 0.603 | 0.049 | 2.251 | Up |
| PKM | P14618-3 | Isoform 3 of Pyruvate kinase PKM | 0.939 | 0.417 | 0.043 | 2.252 | Up |
| CLU | H0YLK8 | Clusterin (Fragment) | 1.396 | 0.616 | 0.008 | 2.268 | Up |
| FZR1 | K7EQT1 | Fizzy-related protein homolog | 1.271 | 0.533 | 0.003 | 2.386 | Up |
| HNRNPA2B1 | P22626-2 | Isoform A2 of Heterogeneous nuclear ribonucleoproteins A2/B1 | 1.724 | 0.708 | 0.046 | 2.434 | Up |
| MROH7 | Q68CQ1-9 | Isoform 5 of Maestro heat-like repeat-containing protein family member 7 | 1.406 | 0.576 | 0.016 | 2.443 | Up |
| TCOF1 | Q13428-8 | Isoform 8 of Treacle protein | 1.397 | 0.568 | 0.007 | 2.458 | Up |
| HNRNPLL | Q8WVV9-2 | Isoform 2 of Heterogeneous nuclear ribonucleoprotein L-like | 1.610 | 0.653 | 0.016 | 2.464 | Up |
| CNPY3 | Q9BT09 | Protein canopy homolog 3 | 1.305 | 0.524 | 0.005 | 2.490 | Up |
| SPAG6 | O75602-2 | Isoform 2 of Sperm-associated antigen 6 | 1.707 | 0.686 | 0.005 | 2.490 | Up |
| TBC1D32 | Q96NH3 | Protein broad-minded | 1.323 | 0.507 | 0.020 | 2.607 | Up |
| GCN1 | Q92616 | eIF-2-alpha kinase activator GCN1 | 1.444 | 0.524 | 0.014 | 2.754 | Up |
| **ATRX** | **P46100-2** | **Isoform 1 of Transcriptional regulator ATRX** | **1.281** | **0.453** | **0.013** | **2.829** | **Up** |
| **JPH1** | **Q9HDC5** | **Junctophilin-1** | **0.810** | **0.253** | **0.025** | **3.207** | **Up** |
| **GRIFIN** | **A4D1Z8** | **Grifin** | **1.456** | **0.437** | **0.039** | **3.333** | **Up** |
| **USP4** | **Q13107** | **Ubiquitin carboxyl-terminal hydrolase 4** | **1.372** | **0. 401** | **0.017** | **3.418** | **Up** |
| **LSAMP** | **F5H5G1** | **Limbic system-associated membrane protein** | **1.378** | **0.403** | **0.017** | **3.418** | **Up** |
| **SERF2** | **C9JQZ0** | **Small EDRK-rich factor 2 (Fragment)** | **0.824** | **0.241** | **0.017** | **3.418** | **Up** |
| **PACS1** | **Q6VY07** | **Phosphofurin acidic cluster sorting protein 1** | **1.379** | **0.378** | **0.045** | **3.654** | **Up** |
| **PDE6C** | **P51160** | **Cone cGMP-specific 3' 5'-cyclic phosphodiesterase subunit alpha'** | **1.303** | **0.345** | **0.037** | **3.773** | **Up** |
| **SLC35C2** | **Q5JW04** | **Solute carrier family 35 member C2** | **0.600** | **0.145** | **0.004** | **4.144** | **Up** |
| **ZNF839** | **A8K0R7** | **Zinc finger protein 839** | **1.814** | **0.362** | **0.050** | **5.005** | **Up** |

Supplementary Table S3 Detailed top 5 canonical pathways.

| Canonical Pathways | P-value | Overlap | Symbol | Up/down |
| --- | --- | --- | --- | --- |
| LXR/RXR Activation | 7.24E-03 | 2.6 %  3/121 | APOH、CLU、ITIH4 | Up |
| FXR/RXR Activation | 8.09E-03 | 2.4 %  3/126 | APOH、CLU、ITIH4 | Up |
| Molybdenum Cofactor Biosynthesis | 1.30E-02 | 2.4 %  3/126 | GPHN | Up |
| Mitotic Roles of Polo-Like Kinase | 1.96E-02 | 3%  2/66 | ANAPC2,FZR1 | Up |
| Acute Phase Response Signaling | 2.07E-02 | 1.7 %  3/179 | APOH,FGG,ITIH4 | Up |

Supplementary Table S4 Top upstream regulators.

| Upstream Regulators | P-value | Target Molecules |
| --- | --- | --- |
| HNRNPA1 | 2.17E-03 | FGG, PKM |
| Recombinant interferon alpha | 3.48E-03 | RNF213 |
| PDE6H | 3.48E-03 | PDE6C |
| cevimeline | 3.48E-03 | HNRNPA2B1 |
| AIPL1 | 6.94E-03 | PDE6C |
| MDC1 | 6.94E-03 | CLU |
| clusterin antisense oligonucleotide | 6.94E-03 | CLU |

Supplementary Table S5 Top network.

| ID | Top Diseases and Functions | Molecules in Network | Score | Focus Molecules |
| --- | --- | --- | --- | --- |
| 1 | [Cancer, Organismal Injury and Abnormalities, Reproductive System Disease] | Actin,Ap1,APOH,ATP6V0A2,BTN2A2,CBFA2T3,CG,CIRBP,CLU,COBLL1,Creb,cytokine,ERK1/2,FGG,GNAS,Growth hormone,HBA1/HBA2,HDL,Histone h3,IgG,Insulin,ITIH4,KIF2C,LTBP4,NLRX1,PACS1,PKM,Pro-inflammatory Cytokine,SLC39A4,SYNE2,TBATA,Tgf beta,TRIP11,Vegf,ZBTB10 | 47 | 21 |
| 2 | [Hematological System Development and Function, Hematopoiesis,Tissue Morphology] | ANAPC2,C2CD4C,CCDC87,CCND1,CD3E,CDK9,CIITA,EPO,ESYT2,FGF8,FZR1,HMOX2,HNRNPLL,HSPA1A/HSPA1B,HSPA4,JPH1,MAD2L1,MROH7,MSH2,NFKBIA,NRAS,NRK,OTUB1,OXR1,p85 (pik3r),PDE6C,PPP2R1A,PTH1R,SERF2,SIPA1L1,SLC35C2,SPP1,TAL1,UBE2N,VHL | 28 | 14 |
| 3 | [Cancer, Endocrine System Disorders, Neurological Disease] | ADAM33,APP,APPBP2,ATAT1,ATP5F1D,CNPY3,EGFR,FLT1,GCN1,HMOX2,HSPA4,Ifn gamma,ITK,LENG9,levodopa,MAP3K7,MCOLN3,NDUFS1,NDUFS6,OTUD7A,RGL1,RNF213,ROBO4,SERAC1,SLC27A3,SPAG6,SPINT2,STAMBP,TMF1,TNFRSF10B,UBC,UBE2N,UNC13B,VEGFB,ZAP70 | 25 | 13 |
| 4 | [Cell Morphology, Nucleic Acid Metabolism, Small Molecule Biochemistry] | ADAMTSL4,ATP5F1D,beta-estradiol,CHD9,CLASP1,CPNE7,DOCK7,FGF8,GPHN,HTT,ID4,ITK,LARP7,LMTK3,LSAMP,MCCC1,MSH2,MTNR1A,NAA30,NDUFS1,NDUFS6,p85(pik3r),PSME4,QSOX1,RBMXL3,SETD2,STAMBP,SUPT5H,TOP3A,TP53,UBE2N,UBR4,USP53,ZNF768,ZNF839 | 23 | 12 |
| 5 | [Cancer, Dermatological Diseases and Conditions, Organismal Injury and Abnormalities] | ADAMTS7,Akt,ATP1B3,ATRX,CD3E,Ck2,FOXD4L1,HNRNPA2B1,Ifn-gamma,IGFBP1,KIAA0319L,KIAA1549,LGALS8,LGALS9,LIMD1,NFkB(complex),p85(pik3r),PCDH15,PODXL,POTEE/POTEF,PRKD2,PTPRG,RNF19B,RPS27A,SLC4A7,SUSD5,TBC1D32,TCOF1,TNFRSF10A,TNFRSF10B,UBTF,USH1C,USP4,VPS13C,ZAP70 | 18 | 10 |
| 6 | [Digestive System Development and Function, Gene Expression, Organismal Survival] | ATP1B1,CFAP20,CHD4,CSNK2B,CUL3,DLG4,EVX2,EYA1,EZH2,FLT1,FOXJ1,FOXP3,HDAC1,HMX1,IFNG,IKZF1,KAT7,KMT2A,LGR6,NEUROD1,NLK,PCDH10,PCDH12,PCDH17,PITX2,PPARG,PPP2R1A,RNF2,SON,TBX2,TBX3,TGFB1,TOP2B,TP53,UBTF | 8 | 5 |
| 7 | [Cancer, Endocrine System Disorders, Gastrointestinal Disease] | BRCA2,C11orf80 | 2 | 1 |

Supplementary Table S6 The categories of the network of cancer

| Categories | Diseases or Functions Annotation | p-value |
| --- | --- | --- |
| Cancer,Endocrine System Disorders,  Organismal Injury and Abnormalities | Thyroid carcinoma | 3.57E-12 |
| Cancer,Organismal Injury and Abnormalities | Head and neck carcinoma | 1.18E-11 |
| Cancer,Organismal Injury and Abnormalities | Cancer of secretory structure | 5.11E-10 |
| Cancer,Organismal Injury and Abnormalities | Incidence of tumor | 2.77E-08 |
| Cancer,Organismal Injury and Abnormalities | Development of carcinoma | 2.95E-08 |

Supplementary Table S7 Top causal network

| Master Regulator | p-value | Target Molecules in Dataset |
| --- | --- | --- |
| IL12 (family) | 1.86E-04 | ADAMTS7,APOH,ATAT1,ATP6V0A2,ATRX,BTN2A2,CBFA2T3,CIRBP,CLASP1,CPNE7,FGG,GCN1,GNAS,HBA1/HBA2,HNRNPA2B1,ITIH4,LGR6,LTBP4,OXR1,PACS1,PKM,PSME4,RNF213,SIPA1L1,SLC35C2,SLC39A4,SYNE2,TCOF1,TMF1,TRIP11,UNC13B,VPS13C |
| genistein | 2.17E-04 | ADAMTS7,ANAPC2,APOH,ATP6V0A2,BTN2A2,CBFA2T3,CHD9,CIRBP,CLASP1,CLU,CPNE7,FGG,FZR1,GCN1,HBA1/HBA2,HNRNPA2B1,HNRNPLL,ITIH4,LGR6,LTBP4,NLRX1,NRK,PCDH15,PKM,PSME4,QSOX1,RGL1,RNF213,SIPA1L1,SLC27A3,SLC35C2,SLC39A4,SUSD5,TBATA,TCOF1,TMF1,TRIP11,VPS13C,ZBTB10 |
| HIP1 | 2.47E-04 | ANAPC2,ATAT1,ATRX,CBFA2T3,CIRBP,CLU,COBLL1,GPHN,HNRNPA2B1,JPH1,LGR6,LTBP4,NRK,OXR1,PACS1,PCDH15,PKM,RGL1,RNF213,ROBO4,SIPA1L1,SLC39A4,TBATA,TCOF1,TMF1 |
| MCF2 | 4.89E-04 | ADAMTS7,APOH,ATAT1,ATP6V0A2,ATRX,CIRBP,CLASP1,CLU,COBLL1,FGG,GNAS,GPHN,HNRNPA2B1,ITIH4,JPH1,LGR6,LTBP4,NRK,OXR1,PACS1,PCDH15,PSME4,QSOX1,SLC35C2,SLC39A4,SYNE2,TCOF1,TMF1,UNC13B |
| SRC | 5.90E-04 | ADAMTS7,ATAT1,ATP6V0A2,CBFA2T3,CLASP1,CPNE7,FGG,FZR1,GCN1,GNAS,HBA1/HBA2,HMX1,HNRNPLL,ITIH4,JPH1,LGR6,LTBP4,NRK,PDE6C,PKM,PSME4,RGL1,ROBO4,SIPA1L1,SLC27A3,SLC35C2,SLC39A4,SON,SYNE2,TBATA,TCOF1,TMF1,TRIP11,UNC13B,VPS13C,ZBTB10 |


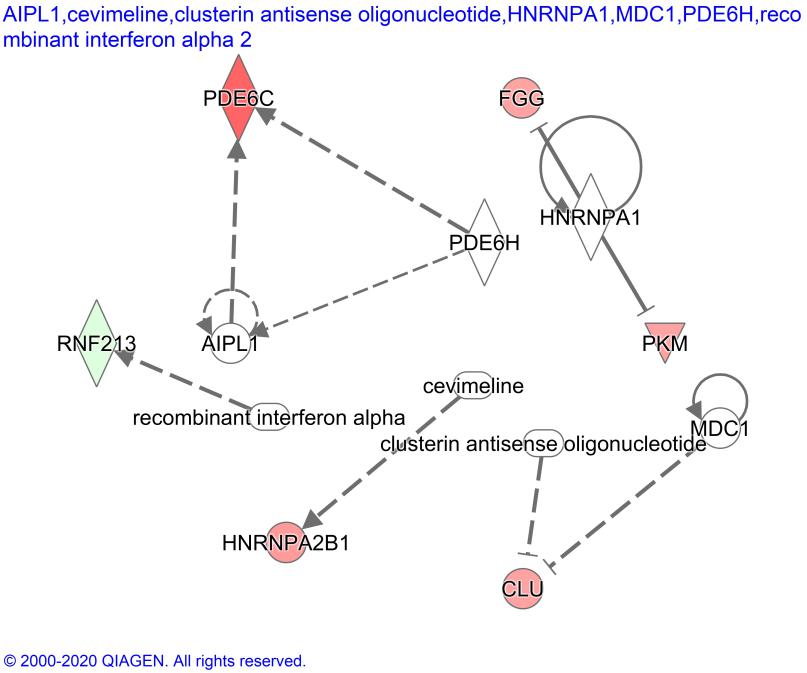


Supplementary Figure S1 The interactive relationships between upstream regulators and target proteins

B

A


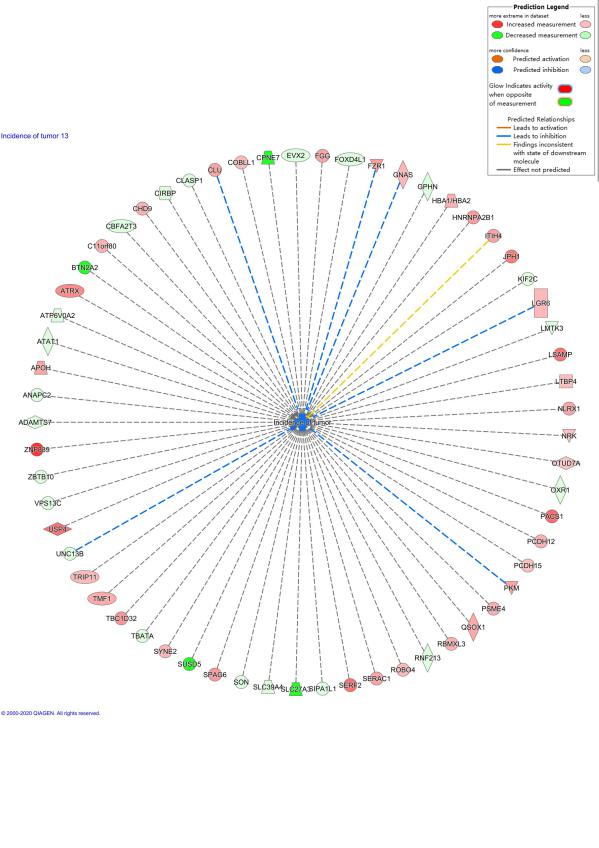

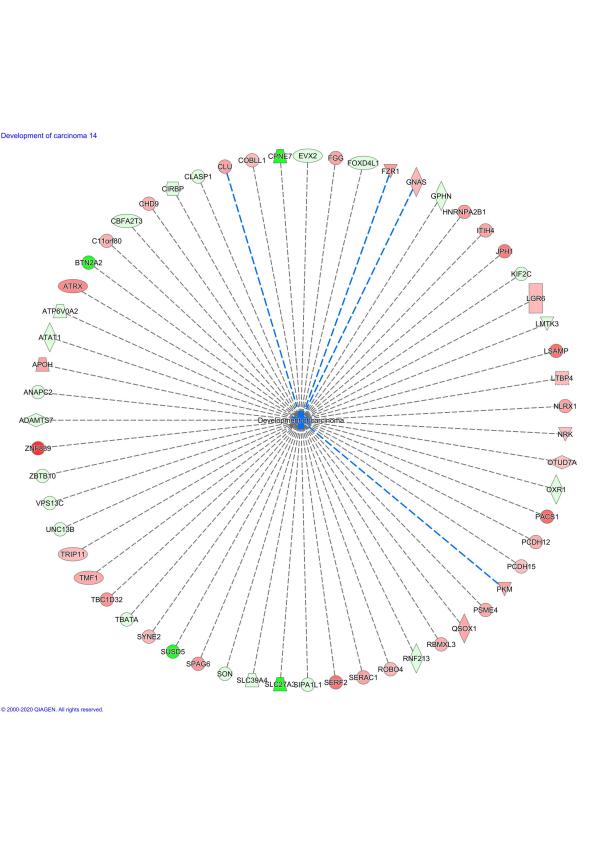


Supplementary Figure S2: The network of incidence and development of cancer: A: Incidence of tumor; B: Development of carcinoma
